# Elevated Neutrophil-to-Lymphocyte Ratio Is Associated With Poor Outcomes for Melanoma Patients Treated With PD-1 Inhibitor or Chemotherapy in a Chinese Population

**DOI:** 10.3389/fonc.2020.01752

**Published:** 2020-09-11

**Authors:** Yalong Qi, Daixiang Liao, Dinglian Mei, Yong Zhang, Yang Liu

**Affiliations:** ^1^Department of Oncology, Beijing Mentougou District Hospital, Beijing, China; ^2^Department of Immunotherapy, Affiliated Cancer Hospital of Zhengzhou University and Henan Cancer Hospital, Zhengzhou, China; ^3^Department of Radiotherapy, Affiliated Cancer Hospital of Zhengzhou University and Henan Cancer Hospital, Zhengzhou, China

**Keywords:** neutrophil-to-lymphocyte ratio, PD-1 inhibitor, melanoma, biomarker, prognosis

## Abstract

**Background:** Previous studies have suggested that an elevated pre-treatment neutrophil-to-lymphocyte ratio (NLR) is associated with worse outcomes in patients with a variety of cancers. The purpose of this retrospective analysis is to investigate the prognostic value of the NLR in a Chinese melanoma population.

**Methods:** Melanoma patients were divided into two groups based on pre-treatment NLR values (≥3 vs. <3). Cox proportional hazard regression analysis and the Kaplan-Meier method were employed to study the prognostic role of the NLR for overall survival (OS) and progression-free survival (PFS).

**Results:** A total of 159 melanoma patients were included in this study, including 40 patients treated with PD-1 inhibitor and 119 patients treated with chemotherapy. In the PD-1 inhibitor group, the median OS was 18.0 months in the low NLR subgroup and 5.6 months in the high NLR subgroup; the median PFS was 7.0 and 2.2 months, respectively. In chemotherapy group, the median OS was 23.0 months in the low NLR group and 8.0 months in the high NLR group, and the median PFS was 9.0 and 4.0 months, respectively. Multivariate analysis showed that the NLR was significantly associated with OS and PFS in melanoma patients treated with either PD-1 inhibitor immunotherapy or chemotherapy.

**Conclusion:** In the Chinese population, an elevated NLR was closely related to worse survival in patients with melanoma treated with either PD-1 inhibitor monotherapy or chemotherapy.

## Introduction

Melanoma is a highly aggressive malignant tumor which occurs most frequently in skin, digestive tract, genitals, nasal cavity and eye. Although the incidence of melanoma is lower in China compared to that in American and European countries, it is growing at an annual rate of 3–5%, and ~20,000 new cases are reported each year (1). In China, the mortality of melanoma also has been increasing rapidly, and most melanoma patients were diagnosed with stage III/IV disease with a really poor prognosis (2). The prognosis of patients with advanced melanoma is poor and the 5-year survival is <5% (3). Furthermore, traditional treatments, such as chemotherapy and targeted therapy, are often ineffective and susceptible to drug resistance. In addition to providing early diagnosis, determining readily, available and reliable biomarkers that can predict patient's prognosis is of great significance for choosing appropriate treatment and improving prognosis.

The clinical application of programmed cell death protein 1 (PD-1)/programmed death-ligand 1 (PD-L1) inhibitors has revolutionarily transformed cancer treatment. Recent studies have demonstrated that a variety of tumors have responses to PD-1/PD-L1 inhibitors. Moreover, if these inhibitors are effective for patients, durable responses can be observed and long-term survival is possible in these populations (4). However, a tremendous limitation of PD-1/PD-L1 inhibitors is the very low response rate (<20%), which means that most patients cannot benefit from the treatment (5, 6). Moreover, these agents have a relatively long median response time, which may cause some patients to miss the optimal treatment window. In addition, although anti-PD-1/PD-L1 antibodies have a low incidence of immune-related adverse events (irAEs), some are serious or even life-threatening (7). Given the low response rate, high cost, and risk of developing rare, yet serious irAEs of novel anti-PD-1/PD-L1 agents, it is important to investigate reliable markers to identify the most suitable patients for such therapy while sparing non-responders from toxicity.

Several predictive markers for anti-PD-1 antibody therapy are currently used in clinical practice, including PD-L1 expression, tumor mutational burden (TMB), and microsatellite instability (MSI). Among them, PD-L1 expression is the most extensively studied. Studies have shown that high PD-L1 expression (tumor proportion score [TPS] ≥ 50%) may be associated with a high response rate and long progression-free survival (PFS) and overall survival (OS) (8, 9). Other studies have reached different conclusions, showing that some patients with negative (TPS <1%)/weak (TPS = 1–49%) PD-L1 expression also have response to PD-1 blockers, whereas some patients with positive PD-L1 expression do not respond to PD-1 blockers (10). Meanwhile, PD-L1 testing requires a biopsy, which is not feasible in some patients. Moreover, PD-L1 expression is biologically heterogeneous and may be modified by previous treatments and different test methods often have different standards (11–14). This marker also has other limitations, such as high costs, and complex procedures. Therefore, alternative markers that are simpler and easier to use, economical and reliable need to be identified.

Accumulating evidence suggests that chronic inflammation plays an important role in cancer progression. As a parameter in routine blood tests, neutrophil is an important indicator of inflammation and has been extensively described in the pathophysiology of autoimmunity and infection. In the cancer setting, the traditional function of neutrophils is being challenged by recent studies, and a novel comprehension of neutrophils has arisen. Emerging evidence indicates that various cancer types often have increased neutrophil infiltration (15). Elevated neutrophils are involved in almost every stage of tumor pathologies, including but not limited to tumor growth, proliferation, invasion, metastasis and angiogenesis (16–18). Lymphocytes, as the body's most important immune cells, play a pivotal role during the antitumour immune response.

The neutrophil-to-lymphocyte ratio (NLR) is closely related to adverse prognosis in patients with various tumors, including lung cancer, colorectal cancer and renal cell carcinoma (19). Although there are also a few studies have been conducted to investigate the prognostic value of NLR in melanoma patients treated with anti-PD-1 antibodies, these studies evaluated the caucasian population, and there are no data from Chinese melanoma patients. There is a big difference between melanoma in China and Caucasians in Europe and America. The differences in genetic etiological mechanisms, pathological morphology, biological behavior, treatment methods and prognosis are quite different (20, 21). Therefore, in order to investigate the relationship between NLR and the response to anti-PD-1 antibody in Chinese melanoma patients, and to investigate the prognostic value of NLR for melanoma patients treated with chemotherapy, we conducted this retrospective study.

## Patients and Methods

We retrospectively analyzed the clinical data of patients with advanced melanoma treated at our hospital between January 2010 and June 2018 and included patients treated with anti-PD-1 antibodies or chemotherapy in this study. The inclusion criteria were as follows: (1) pathologically confirmed melanoma; (2) at least 2 cycles of chemotherapy or anti-PD-1 antibody therapy; (3) at least 1 evaluable lesion; (4) available pretreatment baseline computed tomography (CT) or magnetic resonance imaging (MRI) data; (5) no uncontrolled infections or hematological diseases; and (6) initiation of PD-1 inhibitor is at least more than 4 weeks from the last treatment.

### Clinicopathologic Variables

Clinicopathologic variables for the patients included age, sex, smoking history, drinking history, body mass index (BMI), pathological classification (mucosal vs. non-mucosal), Eastern Cooperative Oncology Group (ECOG) performance status (PS) score, treatment plan (chemotherapy or PD-1 inhibitors), and NLR. NLR was calculated by dividing the absolute number of neutrophils by the absolute number of lymphocytes obtained from a peripheral complete blood cell count. Complete blood cell counts were obtained from all patients within 3 weeks of therapy administration. According to *Law of the People's Republic of China on the Protection of the Rights and Interests of the Elderly*, we defined patients aged 60 years and older are the elderly. In this study, we used 3.0 as the cut-off value for NLR based on the median and mean of NLR and results reported in previous studies (22, 23).

### Efficacy Evaluation

Efficacy was evaluated based on the Response Evaluation Criteria in Solid Tumors 1.1 (RECIST 1.1) (24). CT/MR examination was performed within 3 weeks before the treatment to assess the patients' baseline condition, and then the assessment was performed every 2 cycles of treatment or whenever patients showed apparent symptoms. OS was defined as the time from the initiation of treatment until death. PFS was defined as the time from the start of treatment to disease progression. Surviving patients and progression-free patients as of the last follow-up were defined as censored.

### Statistical Analysis

The Pearson Chi-square or Fisher' exact test was used to assess the association of baseline NLR with clinical characteristics. OS and PFS were conducted and compared using the Kaplan-Meier method and the log-rank test. The prognostic value of each variable was evaluated with univariate Cox proportional hazards model and the significant univariate factors (*P* < 0.1) were included into multivariate. Statistical analysis was performed with SPSS version 21.0 (IBM Corp, USA). All statistical tests were two-sided, and a *P* ≤ 0.05 was considered as a statistically significant difference.

## Results

### Characteristics of the Patients

The characteristics of the patients with melanoma are shown in Table 1. A total of 159 patients were included in this retrospective analysis, including 76 males and 83 females. Most patients (76.1%) were diagnosed as non-mucosal melanoma, and 23.9% were diagnosed as mucosal melanoma. A large majority (81.1%) of patients presented with stage IV disease. Most patients received chemotherapy, and only a small minority (25.2%) of patients received PD-1 inhibitor therapy. We divided the patients into the high-NLR group (NLR ≥ 3; *n* = 47) and the low-NLR group (NLR <3; *n* = 112). No significant differences were observed in baseline data, such as sex, age, smoking history, drinking history, BMI, ECOG PS, stage, anatomic location or treatment, between the high-NLR group and the low-NLR group (Table 1).

**Table 1:** Patient characteristics and association between NLR and clinical categorical variables.

**Variable**	***N*** **(%)**			***P-*value**
	**Overall, *N* = 159**	**NLR <3, *N* = 112**	**NLR ≥ 3, *N* = 47**	
**Gender**				
Male	76 (47.8)	48 (42.9)	28 (59.6)	0.054
Female	83 (52.2)	64 (57.1)	19 (40.4)	
Age (years)				
<60	91 (57.2)	67 (59.8)	24 (51.1)	0.308
≥60	68 (42.8)	45 (40.2)	23 (48.9)	
**Smoking history**				
Yes	38 (23.9)	25 (22.3)	13 (27.7)	0.471
No	121 (76.1)	87 (77.7)	34 (72.3)	
**Alcohol-drinking history**				
Yes	41 (25.8)	25 (22.3)	16 (34.0)	0.123
No	118 (74.2)	87 (77.7)	31 (66.0)	
**BMI (Kg/m**^**2**^**)**				
<24	74 (46.5)	49 (43.8)	21 (44.7)	0.914
≥24	85 (53.5)	63 (56.2)	26 (55.3)	
**ECOG PS**				
0–1	116 (73.0)	82 (73.2)	34 (72.3)	0.910
≥2	43 (27.0)	30 (26.8)	13 (27.7)	
**Stage**				
III	30 (18.9)	25 (22.3)	5 (10.6)	0.086
IV	129 (81.1)	87 (77.7)	42 (89.4)	
**Anatomic location**				
Mucosal	38 (23.9)	30 (26.8)	8 (17.0)	0.188
Non-mucosal	121 (76.1)	82 (73.2)	39 (83.0)	
**Treatment**				
Chemotherapy	119 (74.8)	85 (75.9)	34 (72.3)	0.638
PD-1 inhibitor	40 (25.2)	27 (24.1)	13 (27.7)	

*NLR, neutrophil-to-lymphocyte ratio; BMI, body mass index; N, number; ECOG PS, Eastern Cooperative Oncology Group performance status; PD-1, programmed death-1*.

### An Elevated NLR Is Associated With Worse OS and PFS

**Figure 1:**
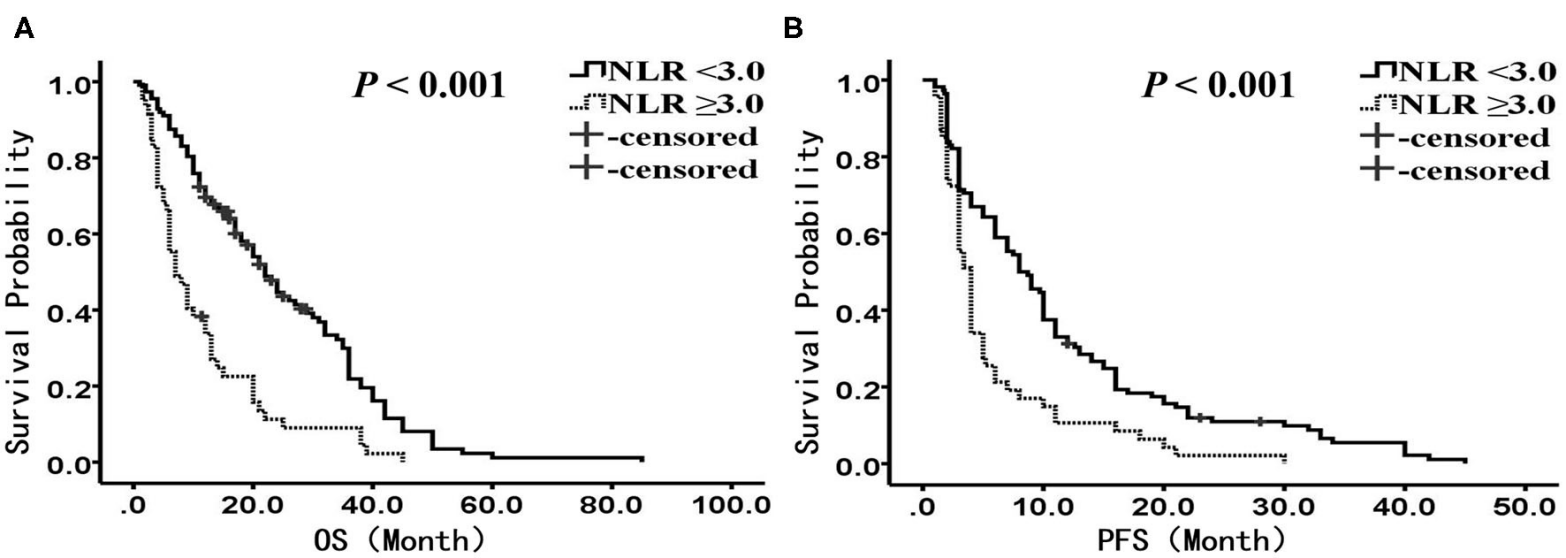
Kaplan-Meier analysis of OS and PFS for the entire cohort. **(A)** OS curve (*P* < 0.001). **(B)** PFS curve (*P* < 0.001). There are 112 patients presented with NLR <3, and 47 presented with NLR ≥ 3. OS, overall survival; PFS, progression-free survival; NLR, neutrophil to lymphocyte ratio.

The median OS was 22.0 months (95% confidence interval (CI): 18.2–25.9 months) in the-low NLR group and only 7.0 months (95% CI: 4.1–9.9 months) in the-high NLR group (*P* < 0.001) (Figure 1A), and the median PFS was 8.0 months (95% CI: 5.7–10.3 months) and 4.0 months (95% CI: 3.4–4.6 months), respectively (*P* < 0.001) (Figure 1B). Univariate survival analysis showed that smoking history, BMI, stage, anatomic location and NLR were related to poorer OS, whereas BMI, stage, anatomic location, treatment, and NLR were related to shorter PFS (Tables 2, 3). In multivariate analysis, NLR was still an independent factor for OS and PFS. The risk of mortality was nearly 2.5 times (hazard ratio (HR): 2.484, 95% CI: 1.706–3.615, *P* < 0.001) higher and the risk of disease progression was 1.9 times (HR: 1.885, 95% CI: 1.300–2.732, *P* = 0.001) higher for patients with elevated NLR (Tables 2, 3). In addition, multivariate analysis showed that smoking history, BMI and stage were also factors affecting OS, and stage was another factor affecting PFS (Tables 2, 3).

**Table 2:** Univariate and multivariate analysis of factors associated with overall survival for all patients.

**Variable**	**Univariate analysis**			**Multivariate analysis**		
	**HR**	**95% CI**	***P***	**HR**	**95% CI**	***P***
**Gender**						
Male	1		0.356	–		–
Female	0.861	0.620–1.197		–	–	
**Age (years)**						
<60	1		0.375	–		–
≥60	1.156	0.829–1.612		–	–	
**Smoking history**						
Yes	1		0.033	1		0.010
No	0.664	0.449–0.981		0.590	0.396–0.881	
**Alcohol-drinking history**						
Yes	1		0.305	–		–
No	0.828	0.569–1.203		–	–	
**BMI (Kg/m**^**2**^**)**						
<24	1		0.001	1		0.001
≥24	1.695	1.215–2.365		1.785	1.264–2.522	
**ECOG PS**						
0-1	1		0.446	–		–
≥2	1.146	0.796–1.649		–	–	
**Stage**						
III	1		0.001	1		0.001
IV	1.935	1.277–2.931		2.030	1.327–3.105	
**Anatomic location**						
Mucosal	1		0.023	1		0.572
Non-mucosal	1.535	1.043–2.260		1.123	0.751–1.681	
**Treatment**						
Chemotherapy	1		0.107	–		–
PD-1 inhibitor	1.445	0.914–2.285		–	–	
**NLR**						
<3	1		<0.001	1		<0.001
≥3	2.528	1.758-3.635		2.484	1.706–3.615	

*NLR, neutrophil-to-lymphocyte ratio; BMI, body mass index; ECOG PS, Eastern Cooperative Oncology Group performance status; PD-1, programmed death-1*.

**Table 3:** Univariate and multivariate analysis of factors associated with progression free survival for all patients.

**Variable**	**Univariate analysis**			**Multivariate analysis**		
	**HR**	**95% CI**	***P***	**HR**	**95% CI**	***P***
**Gender**						
Male	1		0.316	–		–
Female	0.854	0.617–1.181		–	–	
**Age (years)**						
<60	1		0.820	–		–
≥60	1.036	0.752–1.427		–	–	
**Smoking history**						
Yes	1		0.105	–		–
No	0.746	0.513–1.084		–	–	
**Alcohol-drinking history**						
Yes	1		0.737	–		–
No	0.942	0.656–1.355		–	–	
**BMI (Kg/m**^**2**^**)**						
<24	1		0.096	1		0.361
≥24	1.291	0.939–1.774		1.163	0.841–1.608	
**ECOG PS**						
0–1	1		0.163	–		–
≥2	0.781	0.541–1.127		–	–	
**Stage**						
III	1		<0.001	1	1.348–3.266	0.001
IV	2.430	1.590–3.714		2.098		
**Anatomic location**						
Mucosal	1		0.030	1		0.267
Non-mucosal	1.477	1.017–2.145		1.245	0.846–1.833	
**Treatment**						
Chemotherapy	1		0.004	1		0.072
PD-1 inhibitor	1.682	1.151–2.457		1.443	0.968–2.152	
**NLR**						
<3	1	1.390–2.822	<0.001	1	1.300–2.732	0.001
≥3	1.981			1.885		

*NLR, neutrophil-to-lymphocyte ratio; BMI, body mass index; ECOG PS, Eastern Cooperative Oncology Group performance status; PD-1, programmed death-1*.

**Figure 2:**
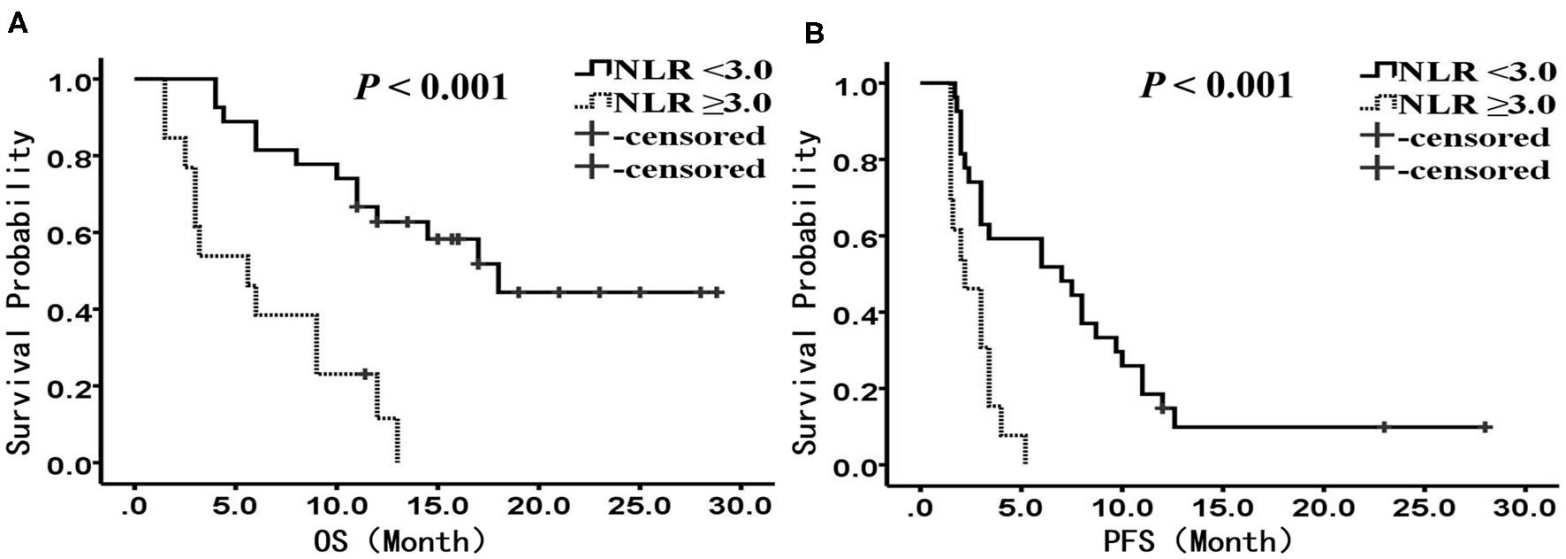
Kaplan-Meier analysis of OS and PFS for patients treated with PD-1 inhibitor. **(A)** OS curve (*P* < 0.001). **(B)** PFS curve (*P* < 0.001). There are 27 patients presented with NLR <3, and 13 presented with NLR ≥ 3. OS, overall survival; PFS, progression-free survival; NLR, neutrophil to lymphocyte ratio.

We performed subgroup analysis to further investigate the prognostic value of NLR in patients receiving different treatments. In the PD-1 inhibitor group (*n* = 40), there were 27 patients with NLR <3.0, and 13 patients with NLR ≥3.0, respectively. The median OS was 18.0 months (95% CI: 12.4–23.6 months) in the low NLR subgroup and 5.6 months (95% CI: 2.1–9.1 months) in the high NLR subgroup (*P* < 0.001) (Figure 2A); the median PFS was 7.0 months (95% CI: 1.8–12.2 months) and 2.2 months (95% CI: 1.0–3.4 months), respectively, with a significant difference (*P* < 0.001) (Figure 2B). Multivariate analysis showed that NLR was the only factor closely related to poor OS and PFS (Tables 4, 5).

**Table 4:** Univariate and multivariate analysis of factors associated with overall survival for patients treated with PD-1 inhibitor.

**Variable**	**Univariate analysis**			**Multivariate analysis**		
	**HR**	**95% CI**	***P***	**HR**	**95% CI**	***P***
**Gender**						
Male	1		0.084	1		0.118
Female	0.506	0.228–1.124		1.751	0.760–4.036	
**Age (years)**						
<60	1		0.614	–		–
≥60	0.816	0.366–1.821		–	–	
**Smoking history**						
Yes	1		0.220	–		–
No	0.578	0.235–1.421		–	–	
**Alcohol-drinking history**						
Yes	1		0.235	–		–
No	0.527	0.178–1.567		–	–	
**BMI (Kg/m**^**2**^**)**						
<24	1		0.291	–		–
≥24	1.522	0.687–3.371		–	–	
**ECOG PS**						
0-1	1		0.030	1		0.255
≥2	2.523	1.050–6.064		1.728	0.674–4.428	
Stage^*^						
III	–		–	–		-
IV	–	–		–	–	
**Anatomic location**						
Mucosal	1		0.216	–		–
Non-mucosal	3.257	0.438–24.226		–	–	
**NLR**						
<3	1		<0.001	1		<0.001
≥3	5.162	2.179–12.226		4.569	1.882–11.091	

NLR, neutrophil-to-lymphocyte ratio; BMI, body mass index; ECOG PS, Eastern Cooperative Oncology Group performance status; PD-1, programmed death-1.

* *All the patients were stage IV*.

**Table 5:** Univariate and multivariate analysis of factors associated with progression free survival for patients treated with PD-1 inhibitor.

**Variable**	**Univariate analysis**			**Multivariate analysis**		
	**HR**	**95% CI**	***P***	**HR**	**95% CI**	***P***
**Gender**						
Male	1		0.550	–		–
Female	1.212	0.630–2.334		–	–	
**Age (years)**						
<60	1		0.610	–		–
≥60	0.849	0.441–1.632		–	–	
**Smoking history**						
Yes	1		0.667	–		–
No	0.853	0.402–1.811		–	–	
**Alcohol-drinking history**						
Yes	1		0.533	–		–
No	0.747	0.288–1.939		–	–	
**BMI (Kg/m**^**2**^**)**						
<24	1		0.579	–		–
≥24	0.837	0.436–1.606		–	–	
ECOG PS				–		–
0-1	1		0.360	–	–	
≥2	1.457	0.628–3.377				
Stage^*^						
III	–	–	–	–		–
IV	–			–	–	
**Anatomic location**						
Mucosal	1		0.110			
Non-mucosal	2.945	0.702–12.358				
**NLR**						
<3	1		0.001	1		0.001
≥3	4.061	1.791–9.210		4.061	1.791–9.210	

NLR, neutrophil-to-lymphocyte ratio; BMI, body mass index; ECOG PS, Eastern Cooperative Oncology Group performance status; PD-1, programmed death-1.

* *All the patients were stage IV*.

**Figure 3:**
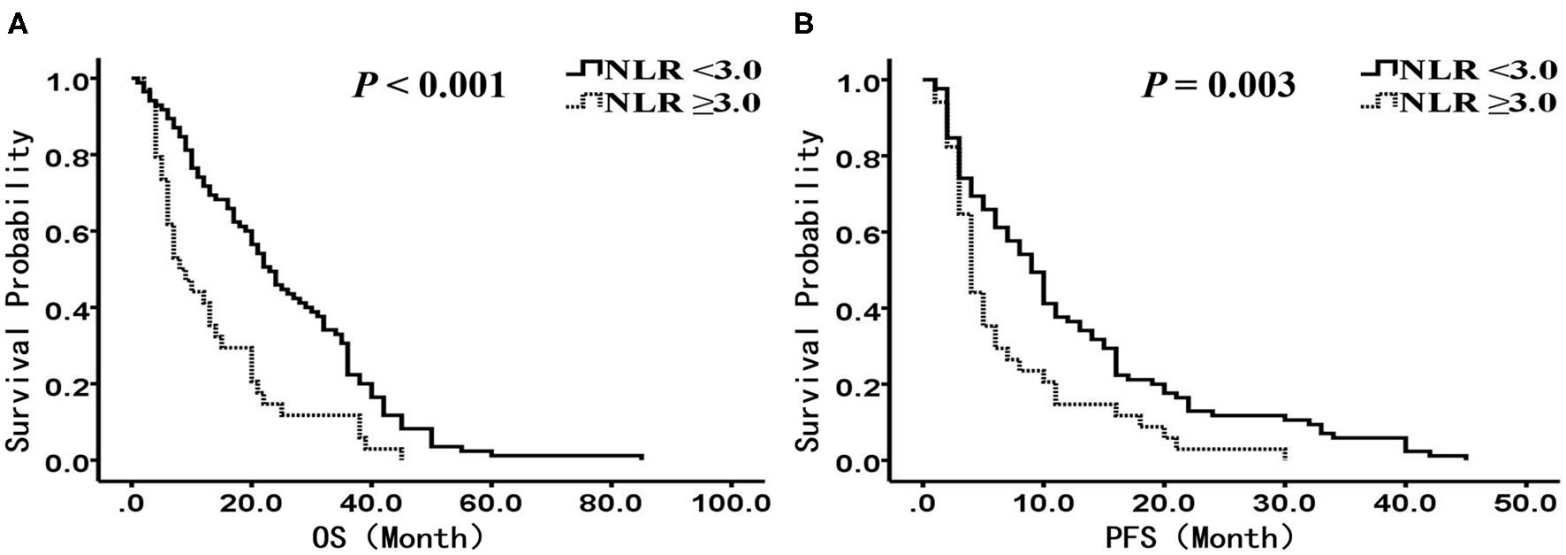
Kaplan-Meier analysis of OS and PFS for patients treated with chemotherapy. **(A)** OS curve (*P* < 0.001). **(B)** PFS curve (*P* = 0.003). There are 85 patients presented with NLR <3, and 34 presented with NLR ≥ 3. OS, overall survival; PFS, progression-free survival; NLR, neutrophil to lymphocyte ratio.

In the chemotherapy group (*n* = 119), there were 85 patients with NLR <3.0, and 34 patients with NLR ≥3.0, respectively. The median OS was 23.0 months (95% CI: 18.5–27.5 months) in the low NLR group and 8.0 months (95% CI: 4.2–11.8 months) in the high NLR group, with a significant difference (*P* < 0.001) (Figure 3A), and the median PFS was 9.0 months (95% CI: 7.1–11.0 months) and 4.0 months (95% CI: 3.2–4.8 months), respectively (*P* = 0.003) (Figure 3B). Multivariate analysis showed that NLR, smoking history, BMI, and stage were independent prognostic factors for OS, while NLR, smoking history, and stage were independent prognostic factors for PFS (Tables 6, 7).

**Table 6:** Univariate and multivariate analysis of factors associated with overall survival for patients treated with chemotherapy.

**Variable**	**Univariate analysis**			**Multivariate analysis**		
	**HR**	**95% CI**	***P***	**HR**	**95% CI**	***P***
**Gender**						
Male	1		0.696	–		–
Female	1.072	0.746–1.541		–	–	
**Age (years)**						
<60	1		0.489	–		–
≥60	0.886	0.615–1.277		–	–	
**Smoking history**						
Yes	1		0.079	1		0.029
No	0.687	0.445–1.063		0.607	0.387–0.951	
**Alcohol-drinking history**						
Yes	1		0.325	–		–
No	0.822	0.549–1.232		–	–	
**BMI (Kg/m**^**2**^**)**						
<24	1		0.002	1		0.002
≥24	1.720	1.192–2.482		1.811	1.233–2.662	
**ECOG PS**						
0-1	1		0.164	–		–
≥2	0.763	0.512–1.136		–	–	
**Stage**						
III	1		0.002	1		0.001
IV	1.893	1.240–2.892		2.055	1.333–3.167	
**Anatomic location**						
Mucosal	1		0.071	1		0.786
Non-mucosal	1.424	0.952–2.129		1.060	0.694–1.621	
**NLR**						
<3	1		<0.001	1		0.001
≥3	2.209	1.463–3.336		2.139	1.392–3.286	

*NLR, neutrophil-to-lymphocyte ratio; BMI, body mass index; ECOG PS, Eastern Cooperative Oncology Group performance status; PD-1, programmed death-1*.

**Table 7:** Univariate and multivariate analysis of factors associated with progression free survival for patients treated with chemotherapy.

**Variable**	**Univariate analysis**			**Multivariate analysis**		
	**HR**	**95% CI**	***P***	**HR**	**95% CI**	***P***
**Gender**						
Male	1		0.158	–		–
Female	1.291	0.887–1.878		–	–	
**Age (years)**						
<60	1		0.968	–		–
≥60	1.007	0.697–1.455		–	–	
**Smoking history**						
Yes	1		0.068	1		0.025
No	0.686	0.446–1.055		0.603	0.388–0.937	
**Alcohol-drinking history**						
Yes	1		0.362	–		–
No	0.838	0.562–1.251		–	–	
**BMI (Kg/m**^**2**^**)**						
<24	1		0.099	1		0.218
≥24	1.334	0.926–1.922		1.264	0.871–1.833	
**ECOG PS**						
0–1	1		0.120	–		–
≥2	0.736	0.488–1.109		–	–	
**Stage**						
III	1			1		<0.001
IV	2.353	1.516–3.652	<0.001	2.323	1.484–3.651	
**Anatomic location**						
Mucosal	1		0.232	–		–
Non-mucosal	1.257	0.844–1.871		–	–	
**NLR**						
<3	1		0.006	1		0.040
≥3	1.775	1.176–2.678		1.551	1.021–2.355	

*NLR, neutrophil-to-lymphocyte ratio; BMI, body mass index; ECOG PS, Eastern Cooperative Oncology Group performance status; PD-1, programmed death-1*.

## Discussion

In recent years, immunotherapy, especially represented by PD-1 inhibitors, has achieved significant advancement, and in certain cancers, immunotherapy has outperformed traditional chemotherapy and targeted therapy (8, 25, 26). In the development of PD-1 inhibitors, melanoma has become one of the first tumor species to undergo clinical trials. These agents have been approved as first-line and second-line treatments for metastatic melanoma based on their significant clinical efficacy. In December 2017, nivolumab was approved as a postoperative adjuvant therapy for lymph node or metastatic melanoma. These clinical data indicate that PD-1 inhibitors are highly effective. However, a significant portion of patients do not benefit from this treatment. Given that PD-1 inhibitor immunotherapy can be associated with a low response rate, significant toxicity and high treatment costs, it is important to investigate predictive biomarkers of the therapeutic response to identify patients who would maximally benefit from such therapy.

Inflammation plays an important role in many pathological processes of tumourigenesis, including but not limited to tumor initiation, proliferation, invasion, metastasis, and angiogenesis (27–29). Neutrophils are the first responders to inflammation, infection, and injury. As one of the most important and abundant types of leukocytes in the immune system, neutrophils play a vital role in these processes (30–32). As a new inflammatory marker, NLR has not been widely used as a prognostic marker, but many studies have shown that an elevated NLR is closely related to inferior outcomes for many tumors, including lung cancer (33), renal cell carcinoma (34), esophageal cancer (35), and breast cancer (36). A meta-analysis of NLR was performed including 40,559 patients with solid tumors, such asgastric cancer, liver cancer, non-small cell lung cancer, pancreatic cancer, breast cancer, soft tissue sarcoma, ovarian cancer, and cervical cancernasopharyngeal carcinoma (37). The result revealed that an elevated NLR was associated with a hazard ratio for OS of 1.81 (95% CI: 1.67–1.97, *P* < 0.001), an effect observed for all disease subgroups, sites, and stages. Hazard ratios for NLR greater than the cut-off for PFS and disease-free survival were 1.63 and 2.27, respectively (all *P* < 0.001). Currently, most studies focus on the relationship between NLR and prognosis after conventional treatments such as surgery and chemotherapy. With the widespread clinical application of PD-1 inhibitors, current data have indicated that NLR has the potential to become a promising prognostic marker.

A retrospective analysis of 97 patients with stage IV melanoma who received nivolumab immunotherapy showed a significant difference in OS (2.9 vs. 16.0 months, *P* < 0.0001) and PFS (2.0 vs. 9.0 months, *P* < 0.0001) between the high NLR group (NLR ≥ 5) and the low NLR group (NLR <5) (38). The response of patients with an NLR <5 was also better than those with an NLR ≥ 5. Multivariate analysis showed that NLR was closely related to OS, PFS and clinical response and that an elevated NLR was a predictor of poor outcomes. In another cohort containing 101 patients with unresectable stage III or IV melanoma treated with anti-PD-1 antibodies, the results showed that along with known prognostic factors, such as lactate dehydrogenase (LDH), PS score, and symptomatic brain metastasis, NLR was an independent prognostic factor for patient outcomes and was closely related to worse survival (39). Similarly, in a recent publication of 224 patients with stage IV melanoma treated with anti-PD-1 immunotherapy (nivolumab or pembrolizumab) as the initial treatment, the baseline NLR was closely related to ECOG PS and the number of metastatic sites, and elevated NLR was associated with lower ECOG PS and more metastatic sites (40). Survival analysis showed that an elevated baseline NLR was independently and significantly associated with an increased risk of death and disease progression in patients with either mucosal melanoma or non-mucosal melanoma, regardless of the mutation status of v-raf murine sarcoma viral oncogene homolog B1 (BRAF) gene. Furthermore, NLR dynamic monitoring showed that an increase in NLR by 30% from baseline after 2 cycles of treatment was also associated with treatment failure and death. Other studies showed that neutrophils were also closely related to tumor ulceration and tumor thickness, both of which were independent negative prognostic factors previously confirmed in melanoma patients (41–43).

To investigate the prognostic value of NLR in Chinese melanoma patients, we conducted this retrospective analysis. In this study, the basic clinical data of 159 patients with advanced melanoma were analyzed. The results indicated that elevated NLR was closely related to worse OS and PFS. The risk of death was nearly 2.5 times higher and the risk of disease progression was 1.9 times higher in the high-NLR group (NLR ≥ 3) compared with the low-NLR group (NLR <3). We further performed subgroup analysis to investigate the relationship between NLR and clinical outcomes of treatment with anti-PD-1 antibodies. Forty patients with stage IV melanoma received anti-PD-1 antibodies after failure of multiple lines of chemotherapy. We analyzed the effect of sex, age, smoking history, drinking history, BMI, ECOG PS, stage, anatomic site, and the NLR on prognosis. Multivariate analysis showed that NLR was the only independent prognostic factor for OS and PFS. The risk of mortality was 4.6 times higher and the risk of disease progression was 4.1 times higher in the high NLR group than in the low NLR group. Surprisingly, we found that an elevated NLR was also an independent prognostic factor in patients receiving chemotherapy and that the risk of mortality was 2.0 times higher and the risk of disease progression was 1.6 time higher in the high-NLR group than in the low-NLR group. To the best of our knowledge, this is the first study to confirm that an elevated NLR is associated with worse clinical outcomes in melanoma patients treated with either anti-PD-1 antibody therapy or chemotherapy in a Chinese population. These results provide strong evidence for the clinical use of NLR as a prognostic marker to optimize patient benefit.

However, the actual mechanism underlying the relationship between an elevated NLR and poor outcomes remains unclear. Several possible explanations have been reported. Melanoma is highly invasive and is prone to metastases. Most patients die due to metastases *via* the lymphatic and circulatory systems. During these processes, neutrophils play an important role. As an important chemokine, C-X-C motif chemokine ligand 5 (CXCL5) is a critical factor in the distant metastasis of various tumors including melanoma, and the role of CXCL5 in tumor metastasis depends on neutrophils (41, 44). CXCL5 expression is significantly increased in patients with advanced melanoma. Compared with melanoma cells with no or low CXCL5 expression, melanoma cells with high CXCL5 expression have significantly greater neutrophil infiltration, suggesting that CXCL5 recruits a large number of neutrophils to melanomas and that the interaction between neutrophils and melanoma cells further promotes tumor cell migration to lymphatic vessels, ultimately leading to lymph node metastasis.

Neutrophils promote not only lymphatic metastasis but also blood metastasis of melanoma. Activated neutrophils have potent adhesion and migration capacities, where β2 integrins play an important role. Intercellular adhesion molecule-1 (ICAM-1) is expressed on the surface of melanoma cells. As a ligand of β2 integrin, ICAM-1 binds to β2 integrin, thereby forming a complex between melanoma cells and neutrophils, which enhances melanoma adhesion to and migration through vascular endothelial cells (45, 46). Moreover, many studies have reported that melanoma can continuously secrete chemokines such as interleukin-8 (IL-8), which upregulates the activity of β2 integrin on the surface of neutrophils, further promoting the binding between ICAM-1 on the surface of melanoma cells and β2 integrin on the surface of neutrophils (47–49). Through this mechanism, neutrophils enhance melanoma adhesion to the vascular endothelial cells, thereby promoting distant metastasis of melanoma *via* blood circulation.

Another important mechanism involves neutrophil extracellular traps (NETs), fibrillar structures, secreted by neutrophils under the effect of various stimulating factors and involved in the innate immune response (50, 51). Many recent studies have shown that in addition to the innate immune response, NETs initiate and promote tumor progression and metastasis *via* several mechanisms (52–54). NETs are composed of depolymerized chromatin and different protein particles, including a loose DNA skeleton, matrix metalloproteinase-9 (MMP-9), neutrophil elastase (NE), cathepsin G (CG), and myeloperoxidase (50). The extracellular matrix (ECM) acts as a barrier during tumor cell invasion and blocks tumor cells from entering the circulatory system. Therefore, degradation of the ECM will facilitate tumor metastasis. As a primary component of NETs, MMP-9 is mainly secreted by neutrophils and plays an important role in ECM remodeling and membrane protein cleavage, promoting cancer cell invasion, migration, metastasis, angiogenesis, inflammation, and proliferation (55–59).

NE is another important protease released by NETs. As a protease implicated in matrix remodeling in various stages of tumor progression, its expression and actively is up regulated in a variety of human tumors, and it correlates with not only disease state but also disease progression (60). NE can activate the extracellular transactivation of membrane receptors such as epidermal growth factor receptor (EGFR) and Toll-like receptor 4 (TLR4), inducing mitogen activated protein kinase (MAPK) signaling and downstream effects, thereby directly promoting cancer cell proliferation. In a mouse model of lung adenocarcinoma [Lox-Stop-Stop (LsL)-K-ras], NE can enter tumor cells to directly induce tumor cell proliferation (61). In addition, NE can indirectly promote tumor cell growth and proliferation by inactivating tumor suppressors, such as Elastin Microfibril Interface Located Protein 1 (EMILIN1) and thrombospondin-1 (62, 63).

NETs can also promote the adhesion of circulating tumor cells to vascular endothelial cells, thereby destroying the integrity of vascular endothelial cells, increasing vascular permeability, and promoting blood metastasis (64–66). The mesh structure of NETs has the capacity to trap tumor cells to form a physical barrier between immune cells and tumor cells, thereby allowing tumor cells to escape surveillance and attack by the immune system (67). These studies indicate that the function of neutrophils is not limited to the processes of inflammation; neutrophils also play a very important role in the stages of tumor origination, growth, proliferation, invasion, metastasis, and immune escape through various complex mechanisms.

The main limitations of this study are related to its retrospective nature and it is a mono-center study. Therefore, there is a risk of bias and confounding factors in patient selection. To control for this, we developed strict inclusion and exclusion criteria, and our results were consistent with those of previous studies. In addition, the sample size of this study was not very large, especially the number of patients receiving PD-1 inhibitor treatment. Third, for objective reasons, we lost some clinical and pathological data, such as LDH, BRAF mutation status, and PD-L1 expression. Last, at present, there is no unified standard for the calculation of the NLR cut-off value. We used a value of 3.0 as the cut-off in this analysis based on our data analysis and previous studies. However, further research is needed to validate this cut-off value. Taking into account these factors, we hope that in the future more prospective, randomized and large-scale clinical trials will be conducted and provide more reliable data.

This study suggests that an elevated NLR is closely and independently related to poor clinical outcomes in advanced melanoma patients receiving either PD-1 inhibitor immunotherapy or chemotherapy. Notably, NLR testing is economic, readily available, non-invasive, biopsy-independent, repeatable, and dynamic. Therefore, NLR may be an ideal prognostic indicator for patients with melanomas. In the future, prospective, multi-center clinical trials are needed to confirm this conclusion, and basic studies are also needed to further clarify the mechanism by which neutrophils promote tumor progression to enable NLR application in clinical practice as soon as possible, helping clinicians to develop more suitable and effective treatment regimens for patients.

## Data Availability Statement

The raw data supporting the conclusions of this article will be made available by the authors, without undue reservation.

## Ethics Statement

The studies involving human participants were reviewed and approved by The Ethics Committee of Henan Cancer Hospital. Written informed consent for participation was not required for this study in accordance with the national legislation and the institutional requirements.

## Author Contributions

YQ and YZ performed the literature search and designed the study. YQ, DL, and DM contributed to the acquisition, analysis, and interpretation of the data. DM and DL contributed to data collection. YQ wrote the original draft of the manuscript. YZ, DL, and YL edited the manuscript. All authors read and approved the final version.

## Conflict of Interest

The authors declare that the research was conducted in the absence of any commercial or financial relationships that could be construed as a potential conflict of interest.
